# Emerging applications of 3D engineered constructs from plant seed extracts

**DOI:** 10.55730/1300-0152.2645

**Published:** 2023-03-14

**Authors:** Sedat ODABAŞ, Bora GARİPCAN, Rihab KSOURI

**Affiliations:** 1 Biomaterials & Tissue Engineering Laboratory (bteLAB), Department of Chemistry, Ankara University, Ankara, Turkey; 2 Interdisciplinary Research Unit for Advanced Materials (INTRAM), Ankara University, Ankara, Turkey; 3 Biomimetic and Bioinspired Biomaterials Laboratory, Institute of Biomedical Engineering, Boğaziçi University, İstanbul, Turkey

**Keywords:** Seed-extract, scaffolds, tissue engineering, natural macromolecules

## Abstract

Seeds, by-products derived from various plants such as mango, quince, and apples, are considered waste, though they have emerging commercial potential, and have been used in biological, industrial, and physiological research. Seed-derived natural macromolecules— mainly polysaccharides, mucilage, gums, and cellulose—have physicochemical and structural diversification, giving the potential for forming gels, texturing, thickening, and providing interfacial adsorption. Seed-derived natural macromolecules have been widely used during the last few years in cell research and tissue engineering applications. Their widespread approachability and safety, high rate of biodegradability, biocompatibility, supporting cell proliferation, and extracellular matrix synthesis are the main properties making plant seed derivatives appropriate for use. The gel-forming ability of these derivatives gives them the capability of creating natural polymer-based scaffolds with the aptitude to resemble extracellular matrices (ECM). These ECM exhibit the high potential in scaffolds for tissue renewal. A deeper knowledge of the physicochemical characteristics of seed-derived mucilage and gum has been indicated as a key ingredient in several pharmaceutical preparations, but it has been remarkably utilized in nanomedicine for the last few years as a drug carrier for drug delivery, in gene therapy, and as scaffold components for tissue engineering purposes. Here, we afford up-to-date data about the different extracts from plant seeds—mainly mucilage and gum, we summarize the extraction techniques used to isolate these macromolecules, and we focus on their application in scaffold fabrication for tissue engineering purposes and regenerative medicine applications.

## 1. Introduction

Mimicking organs’ microenvironment and providing the adequate structure and necessary support for cell growth and differentiation remains an enormous challenge in tissue engineering. The purpose of tissue engineering persists in allowing the necessary physical and biochemical properties of tissues. These modifications allow cells to spread and attach, prompting the expression of specific genes and providing mechanical and chemical cues based on the scaffold (Nair and Laurencin, 2007; O’Brien, 2011; Camci-Unal et al., 2014, Ramos and Moroni 2020). Scaffolds provide the structural and mechanical framework for tissue growth in the lab, closely mimicking the native 3D microenvironment. Some scaffolds are made from highly porous biological materials, which provide suitable support for cells to attach to and grow. Applications of new bio-based scaffolds seeded with stem cells can develop, specialize, and ultimately replace damaged or missing tissue. Bio-based materials as matrices in tissue regeneration allow the regulation of framework, and mechanical behavior. Besides, they offer a morphology and bio-interfaces as surrogates that mimic native extracellular matrix (ECM) system, which is composed of several macromolecules and offers physical support, communication pathways, and 3D organization to the cells in organs and tissues (Nair and Laurencin, 2007; O’Brien 2011; Camci-Unal et al., 2014).

Plants are widely used nowadays because they have a high surface area, water transport properties, vascular networks, and an extensive range of mechanical properties. Thanks to their bioactive components, they remain the best alternative as tissue scaffold instead of animal-based uses, diminishing the ethical concerns of animal use; besides, they are facile to access with the minimum cost (Song et al., 2018; Bilirgen et al., 2021). Plants own a plethora of diverse mechanical properties such as cohesiveness and elasticity, provided the most by flexible and tough components such as leaves and seeds. However, these components are still considered waste.

According to data provided by the Food and Agriculture Organization (FAO), around 50% of fruit production is composed of seeds and peels, and they are considered food wastes (Villacís-Chiriboga et al., 2020). Dicing of papaya produces about 6.5% of seed waste, apples generate 10.91% of seed while the processing of mangoes produces about 13.5% of seeds (Ayala-Zavala et al., 2010). It is important, though, to minimize the environmental impact of seed wastes derived from fruits and vegetables, such as the production of biogas electricity, the formation of the environment, and air pollution (Aktaş et al., 2012)

Yet, recovering their beneficial compounds and their conversion to other useful biomass and bio-products are limited. Nevertheless, fruit wastes make up the niftiest candidate because they are rich in biodegradable organic constituents (Baliga et al., 2011).

Seeds as by-products are discarded or partially incorporated into animal feed. Their valuation in industrial applications or in the construction of scaffolds remains very little explored, apart from a few traditional applications such as phytotherapy and as excipients in pharmaceutical formulations such as gels and tablets (Mustapha et al., 2015). The seeds of fruits contain extractable components with high added value. Besides being rich in protein, dietary fiber, phenolic compounds, and antioxidants (Hamada et al., 2002; Baliga et al., 2011), seed extracts are mainly polysaccharide bases, with a form of mucilage, glucan, and gums common in their amorphous and monosaccharide composition. Mucilage and gums are also hydrophilic molecules, forming gel or a viscous solution with water. Those forms are advantageous for their biodegradability, exemption from toxicity, and biocompatibility (Soukoulis et al., 2018; Waghmare et al., 2022).

**Figure 1:**
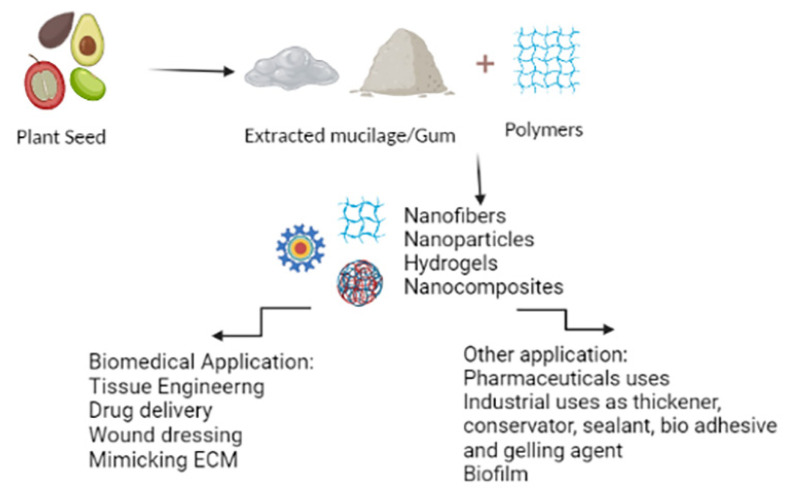
Various applications of plant seed-derived extracts (created by Biorender).

Given their richness in bioactive compounds, researchers investigated plant seeds in several disciplines including industrial, beverages, and pharmaceutical areas as a thickener, conservator, sealant, bioadhesive, and gelling agent, making them a strong tool to take benefits (Figure 1) (Soukoulis et al., 2018). However, the fabrication of biocompatible, eco-friendly, and smart advanced biomaterials from natural systems has emerged as one of the most auspicious approaches for scaffold development (Xue et al., 2018). Seed extracts have taken an outstanding part in scaffold assembly during the last decades. Their wide application is due to their complex organization from the cellular to the macroscopic scale and their ability to mimic the extracellular matrix environment. They also have shown a remarkable role in drug delivery, gene therapy, and the synthesis of biological compounds (Šola et al., 2019; Amiri et al., 2021).

This review highlights recently published work related to the usage of plant seed extracts, mainly polysaccharides, as a blend component to develop biocompatible and efficient biomaterials for tissue regeneration. We summarized the mainly used sources of available seeds, the extraction techniques of their active biomolecules, and the key properties related to these extracts to develop tools for regenerative medicine applications.

## 2. Methodology

Terms such as plant, seed, extract, gums, mucilages, scaffold, and tissue engineering were used to search methodology from scientific databases, including Scopus, ISI Web of Knowledge, PubMed, and Google Scholar.

The collective research of original papers and reviews, including mucilage and seed terms alone or associated with scaffold was followed up to identify the emergence of uses of those candidates and in which areas.

**Figure 2:**
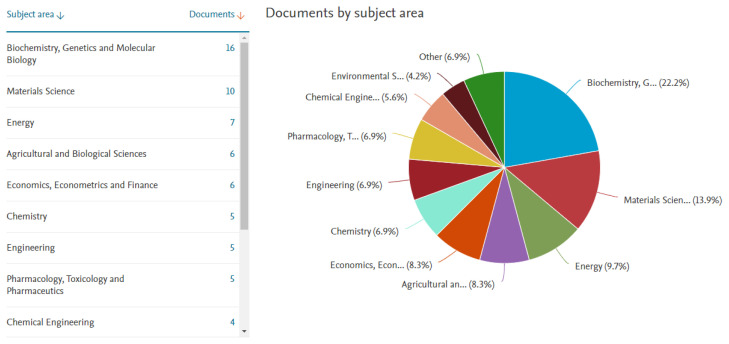
Worldwide research and application area of mucilage for scaffold preparation. Updated on 07/06/2022.

The results of this screening, as shown in Figure 2, demonstrate the different application areas of seed-derived mucilage using mucilage and scaffolds as keywords. As it is clear, the use of seed mucilage is considered a new technology developing in the recent few years for different purposes. The main application area of these extracts seems to be related to biochemistry and molecular biology; besides, materials science is the second large area of mucilage application.

## 3. Plant-seed macromolecules extraction and general applications

Seeds are considered the embryonic form of plants. They are covered by an outer protective layer and rich in reserves or extracts such as protein, carbohydrates, and oil (Petrů, 2017). Seeds have a design and a composition allowing them to gain original properties to be a stronger tool than the sum of a plant’s constituents. Extraction and purification are essential for processing (El Sheikh, 2014). The constant petition to extract bioactive fractions from plants encourages perpetual searches to select the most convenient extraction processes. Hence, the extraction method usually defines the quality of the produced material, its efficacy, and its increasing impact on the yield of the extract. Affecting parameters such as temperature, pH, time, and the techniques applied for the isolation also affect the extract quality and yield. Plant seed mucilage can be extracted directly by soaking the intact seed or the seed husk in water due to its extremely hydrophilic nature, and this is considered the gold standard for mucilage extraction from seeds according to the literature (Izadyari et al., 2020; Simsek et al., 2020; Amiri et al., 2021; Guzelgulgen et al., 2021; Yilmaz et al., 2021).

Numerous extraction methods of seed’s bioactive compounds are to be mentioned, and they can be divided into conventional methods and eco-friendly ones known as nonconventional ones. Briefly, chemical, and enzymatic methods are used. Besides physical methods such as ultrasound, and pulsed electric fields, microwave heating and supercritical fluids are used for bioactive compound extraction from plant material (Heinz et al., 2003; Ghafoor et al., 2010; Ghafoor et al., 2011; Simsek et al., 2020). The techniques used to extract mucilage and gums from some seed species are summarized in Table 1.

The uses of water as a solvent for bioactive molecules extraction is generally followed by heat treatment, e.g., for mucilage extraction from chia seed, both cold and hot methods were used to evaluate the role of temperature on mucilage yield (Tavares et al., 2018). Sometimes, this method is not sufficient for mucilage separation; other methods are needed in this case, such as the usage of chemical, enzymatic, or physical methods to dissociate the nonhydrolysable part of the mucilage (Zhao et al., 2017).

The extraction using enzymatic or mechanical forces may be harmful, and the extract may lose its own structure, hence the need to use other methods. Extraction with the ethanolic method is the second most widely used method for the seed content extraction and precise mucilage (Shekarabi et al., 2014; Cheirmadurai et al., 2016) while other studies used rotary evaporator (Bal and Swain, 2020). Some studies combine all the methods at once, such as Renfrew et al., who reported a novel protocol using ethanol and ether separately or combined, followed by extraction with water. The precipitation of mucilage was achieved then with alcohol (Abbastabar et al., 2015).

Many studies have confirmed the arrangement and the stabilizing of part of the mucilage and fractionated plant seeds. The role of the seed mucilage extract has been demonstrated on a physiological scale after ingestion through the gastrointestinal tract, which has been shown to have many benefits for health such as the modulation of the glycemic response, insulinemia, increasing satiety, and regulating the function of gut microbiota (Kay et al., 2017). Seed mucilage and gums are broadly used for forming biofilm, as crystalizing mediators, and for wound dressing in the medical and pharmaceutical industries, as well as in the food industry as gelling agents, stabilizers, emulsifiers, and thickeners (Soukoulis et al., 2018).

## 4. Plant seed extracts as potential source for biomedical scaffolds

Generating a suitable environment for tissue regeneration is based on the use of physical constructs with interconnected pores that have the ability to act as a niche for cell adhesion, migration, proliferation, and differentiation (Robu et al., 2011). Plant seeds are considered one of the most potential physical constructs, containing a variety of polysaccharides that differ in their physiological function, such as cellulose, hemicelluloses, pectin, gums, mucilage, and starches, which differ in their chemistry, physiological function, sources, and application (Table 2). According to Pereira et al. (2016), extracted oil or mucilage from fruit seed was demonstrated to contain biological macromolecules and bioactive materials capable of regenerating dermal-ECM with an acceleration of the wound healing process (Pereira and Bártolo, 2016).

### 4.1. Scaffolds from seed-derived mucilage

One of the major outcomes of a plant’s metabolism is mucilage. Mucilage is usually produced by higher plants like shrubs and flowering herbs. It belongs to the carbohydrate family with an elevated rate of solubility in water, acting as a binding, thickening, and stabilizing agent (Waghmare et al., 2022). Besides, mucilage is known to be a common tool for food packaging, as an adjuvant for pharmaceutical preparations, and is used also in the cosmetic industry (Waghmare et al., 2022). Its use in regenerative medicine has been shown in a few studies as an alternative to synthetic polymers, nanocomposites, or as nanocarriers (Hajivand et al., 2019; Yavari Maroufi and Ghorbani 2021). Several natural sources of plant mucilage seeds have been mentioned in the literature with different origins, such as seeds from flax, yellow mustard, basil, chia, and quince (Soukoulis et al., 2018).

Quince seed extract has been largely investigated for its potential use in tissue engineering and developing hydrogels due to its high content of cellulose and hydrolysable polysaccharides, here its mucilage, which have been shown to have various biological effects, including improvement in mechanical properties, the ability to stimulate cell proliferation and promote tissue repair (Tosif et al., 2021).

**Figure 3:**
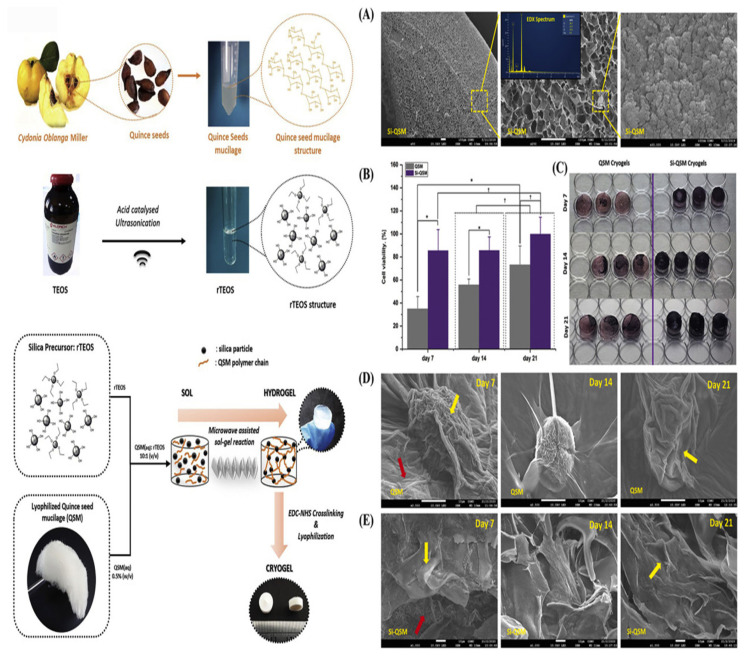
The steps to extract mucilage quince seeds by preparation of silica precursor for developing Si-QSM based cryogels with microwave-assisted technique. Figures A, B, C, and D show the examination of cryogel morphology with SEM microscopy with of hAMSCs attachment on QSM cryogel and Si-QSM cryogel on days 7, 14, and 21. Reproduced, with permission, from Yilmaz et al. (2021).

A recent study by Guzelgulgen et al. (2021) utilized quince seed-derived glucuronoxylan based hydrogel (QSH) as a scaffold (Figure 3) with highly porous and interconnected structure, swelling capacity, and high biocompatibility which are a fundamental issue in scaffold design demonstrating its strong role as a candidate for 3D cell culture (Guzelgulgen et al., 2021). The mechanical strength was defined through AFM analysis to measure the surface topography, force-distance profiles, and Young’s modulus values. Samples were shown to explore elastic properties by being indented at rates of approximately 1 −m s^−1^, proven to be the closest for viscoelastic properties of cells, matrix, or substrates.

The gelation procedure was conducted by varying the concentrations of both QSH and the cross-linker, which in this case was glutaraldehyde (GTA). NIH-3T3 mouse fibroblasts were cultured to approve the biocompatibility of QSH and results showed that the best viability and proliferation of cells were obtained at 2 mg/mL of QSH. Here, the authors prove the enhancement of ECM microenvironment through the upregulated expression of collagen I, the primary protein of the ECM, besides, the electrostatic interaction between growth factors and other ECM proteins were associated with the anionic GAGs sourced from quince seed gum (QSG), pectin, and nanocrystalline cellulose (Guzelgulgen et al., 2021), stimulating the mechanical forces, and promoting tissue formation.

Allafchian et al. (2021) combined polyvinyl alcohol (PVA) with synthesized Balangu (Lallemantia royleana) seed mucilage (BSM) solutions for the purpose of producing 3D electrospun cell culture scaffolds. For this purpose, they integrated PVA into BSM nanofibers, which facilitated the formation of the scaffolds. The developed structures’ mechanical strength was approved through improved physiochemical characteristics such as increased electrical conductivity, viscosity, and surface tension tests (Allafchian et al., 2021). In another study, the hydrocolloid quince seed mucilage was enriched with needle-like nano-hydroxyapatite (nHAp) crystals to fabricate a novel biomimetic osteogenic scaffold (Cetin Genc et al., 2022). The characteristics of developed structures were evaluated by Fourier-transform infrared spectroscopy (FTIR), X-ray diffraction (XRD), scanning electron microscope (SEM), and elemental mapping. Swelling and enhanced mechanical durability were evaluated in favor of hydrocolloid backbone of the developed structures, which offered good features for osteogenic development. Moreover, the EDC/NHS crosslinked QSM-nHAp scaffolds had higher mechanical durability than noncrosslinked scaffolds.

In another study by Warrand et al. (2005), *Linum usitatissimum* L. seed mucilage was extracted, and an investigation of its heterogeneity was made. After fractionation, the analyses of the neutral monosaccharide’s composition showed an assortment of three main polymer families with remarkable rheological properties, providing different networks to form gels (Warrand et al., 2005).

Yilmaz et al. (2021) used microwave-assisted sol-gel reactions to prepare cryogels, composed of silicon and quince seed mucilage (Si-QSM) (Yilmaz et al., 2021). EDC/ NHS was used as a cross linker for fabricated QSM cryogels and as a control for Si-QSM. Mechanical tests were made to study the mechanical properties of the products, besides their porosity, swelling rate, and an enzymatic biodegradation test was realized. All analyses confirmed that Si-QSM scaffolds have the feature of an interconnected porous network around 233 ± 40 −m and 64 ± 15 −m which is believed to be the optimal pore size for idyllic waste exclusion and nutrient supply according to the literature for bone tissue regeneration (Chen et al., 2018).

The evaluation of the cellular hosting capabilities of the cryogels for osteogenic induction was made through field emission-scanning electron microscopy (FE-SEM). FE-SEM results showed a well-defined spatial edge for cell behavior control such as proliferation, differentiation, and hosting of the engineered 3D microstructure. Furthermore, fabricated scaffolds showed a high capacity for swelling, which boosted cellular hosting and proliferation (Yilmaz et al., 2021). Human adipose mesenchymal stem cells (hAMSCs) were used as model cells to assess osteogenic differentiation and osteobiologic performance. Biological behavior and biomineralization have been shown to be enhanced on the fabricated cryogels. MTT results proved the nontoxic behavior of both cryogels as well as the increased metabolic activity within 21 days through supporting cell attachment and proliferation substantially. Gene expression analysis demonstrated that the osteobiologic capacity of Si-QSM cryogels showed an upregulation of the collagen type 1 α1, Osteonectin, and Runx transcription factor 2 genes expression up to day 21 on hAMSCs (Yilmaz et al., 2021). In addition, alkaline phosphatase expression was modulated in both QSM and Si-QSM cryogels, showing their capabilities of repair and regeneration, though, as a tool of future use in bone research studies. Structures of pores shown in Figure 3 after SEM microscopy micrographs were continuously covered with silica molecules believing that it is the ideal size for waste removal and nutrient supply in vivo. Besides, a better interaction of the cells with the cryogel, enhanced cell attachment, and boosted cell proliferation were observed in the silica-modified structure of fiber-like QSM.

Another study used a microwave-assisted technique for the construction of a polymer from fenugreek seed mucilage (FSM) combined with PVA and polyacrylamide (FSM-PVA-g-PAM) for drug delivery purposes (Bal and Swain 2020). The characterization of the synthesized graft copolymer demonstrated that the intrinsic viscosity leads to a better swelling capacity and late drug release, and this is related to the long polymeric chains. Tissue growth in vitro on the graft copolymer sample was effective, and histopathological studies confirmed the nontoxicity of the constructs.

Şimşek et al. (2020) fabricated a 3D porous construct from quince seed mucilage (QSM) to study the behavior of hAMSCs (Simsek et al., 2020). Cross-linked scaffolds from QSM showed an interconnected and extremely porous construction in the assimilation of the noncrosslinked scaffolds. The assessment of cell viability of hAMSCs with the cross-linked QSM-based scaffolds revealed that the scaffolds are exempt from any cytotoxic effect, representing a strong tool for cell proliferation and adherence and for tissue engineering applications.

The high-water content of quince mucilage and the exposition to infections of open wounds gave the idea of developing scaffold-based QSM extract modified by Polycaprolactone -Polyethylene glycol (PCL-PEG) copolymer (Izadyari Aghmiuni et al., 2020). A comparison was also made between the properties of this scaffold and those of four other scaffolds. The difference was followed up by the variation of the ratio of PCL/PEG, or with changing the QSM with PCL-Chitosan-PEG, with chitosan alone, or just QSM, to evaluate the improvement in the engineered skin scaffolds based on the QSM and its combined effect with polymers. The solutions of different polymers with the QSM were prepared and cross-linked with the NHS/EDC crosslinking solution. In vitro studies were conducted to mimic the mechanobiological behavior of skin, to assure the promotion of fibroblast spread and the differentiation of hAMSCs into keratinocytes (Izadyari Aghmiuni et al., 2020). Routine analyses of scaffolds were conducted, such as swelling test, degradation rate, porosity, and mechanical and biological behavior. Results demonstrated that PCL/QSM/PEG polymer possesses remarkable physicochemical properties giving it the role of a stimuli-responsive biomatrix allowing fibroblast growth and promoting hAMSCs adhesion. This polymer increases the mechanobiological signals which induce keratinocytes for wound healing. This study reveals that QSM can be a suitable applicant for wound dressings and skin tissue engineering as inducing biological factors.

**Figure 4:**
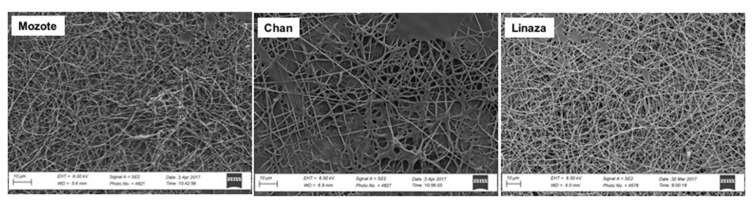
SEM images from fibroblasts (L929) culture experiment and surface examination of the biocompatibility of nanofibers of mucilage from Mozote, Chan, and Linaza with PVA. Cell proliferation on mucilage/PVA NFs was significantly higher than that on PVA NFs, with better proliferation in the presence of chan seed mucilage. Reproduced, with permission, from Urena-Saborio et al. (2018).

Chan (*Hyptis suaveolens*), linaza (*Linum usitatissimum*), and mozote (*Triumfetta semitriloba*) are at the center of the development of mucilage-based beverages in America given their wholesome effects. Chan beans, linaza beans, and mozote seed-derived mucilage were used to fabricate electrospun nanofibers (ESNFs) in combination with PVA (Urena-Saborio et al., 2018). Results prove that seed-extracted mucilage augmented the proliferation of fibroblasts, and this suggests that all mucilage presence, with the ESNFs and PVA, is meaningfully more improved than PVA-NFs alone, and it is appropriate for cell growth. L929 fibroblast adherence, spreading, and better growing were visualized by SEM images with a large surface area of the extract (Figure 4). This study proved that mucilage/PVA ESNFs are a good tool for tissue engineering applications while this mix can act on fibroblasts by enhancing their biocompatibility and attachment (Urena-Saborio et al., 2018). Results show plant mucilage-based ESNFs are well-suited for fibroblast cell growth, significantly better than ESNFs of PVA, and the mucilage of chan beans is better than that of mozote and linaza for supporting cell proliferation.

Another study has focused on preparing an electrospun scaffold in combination with basil seed mucilage (BSM) and polycaprolactone (PCL) and focusing on whether the composition is suitable for cell growth (Allafchian et al., 2018). The two polymers were combined in different concentrations and then electrospun. The polymer in this study has a high porosity of around 90% and 25% degradation rate, which was reached after 4 weeks. Based on the test used to determine the hydrophilicity of the scaffold, adding BSM to the polymer solution significantly raises the hydrophilicity of the scaffold, which improves cell adhesion. The appropriate ratio for cell culture studies was found to be 3/2 of PCL/BSM (PCL 10% and BSM 2%) on which Vero epithelial cells show better adhesion and growth.

### 4.2. Scaffolds from seed-derived gums

Gums are known to be a vicious secretion form of some plants that is soluble in water and takes on a rigid form after drying. They are basically carbohydrates linked to proteins and minerals in their construction and have been identified as sustainable, biodegradable, and biosafe bioconstructs. Gums may be derived from different sources, mainly leaves, bark, and seeds (Amiri et al., 2021)**.** Amine et al. (2007) extracted gum from durian seed, then this method was adjusted where the chemical compositions of durian seed-derived gums, and their rheological and viscoelastic behavior were characterized (Amid and Mirhosseini, 2012). Gum possesses diverse properties of attracting water giving them high viscosity and swelling properties and its ability to form a biofilm (Mohammadinejad et al., 2020). The application of gum-derived seed was studied in the gastrointestinal tract through drug release (Zemzmi et al., 2020), and on colorectal cancer as antitumor medications (Chaurasia et al., 2006).

Recently, physicochemical, and functional characteristics have been studied in various plant gums where they perform many structural applications. The gum was extracted from *Cassia javanica*, *Caesalpinia pulcherrima* (Singh et al., 2009), mucilage from *Linum usitatissimum L.* seeds (Warrand et al., 2005) and gum from Mesquite seed (Estévez et al., 2004), besides functional properties and rheology of the biopolymer from the heteropolysaccharide and protein from *Durio zibethinus* seed were studied (Amid and Mirhosseini, 2012).

**Figure 5:**
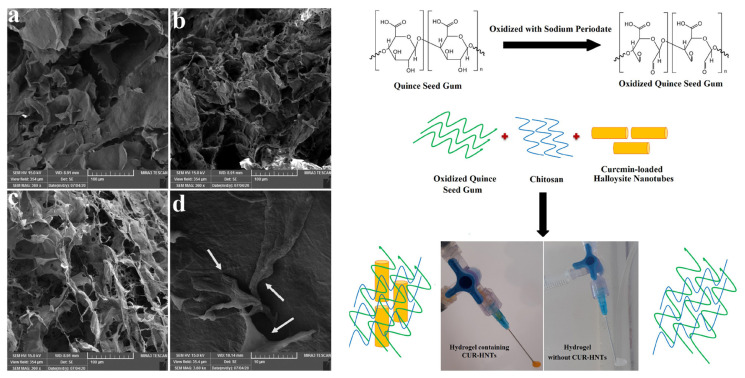
(Left) Oxidized quince seed gum (OX-QSG) preparation and formation of Chitosan/OX-QSG/halloysite nanotubes (CS/OX-QSG/ HNTs) hydrogels. (Right) Scanning electron microscopy (SEM) images of Chitosan/Oxidized Quince Seed Gum (CS/OX-QSG) hydrogels with different content ratios of CS/OX-QSG (a) 75:25, (b) 50:50, (c) 25:75, and (d) SEM images of NIH3T3 fibroblast cell attachment. Reproduced with permission from Yavari Maroufi and Ghorbani 2021.

Maaroufi et al. (2020) developed a nanocomposite to improve hydrogel based on the mix of a natural polymer of chitosan, gum from quince seed modified by oxidation (OX-QSG), and curcumin-loaded halloysite nanotube (Figure 5) to form nanocomposite hydrogels composed of chitosan with oxidized quince seed gum and halloysite nanotube loaded with curcumin (CS/OXQSG/CUR-HNTs) (Yavari Maroufi and Ghorbani, 2021). Besides, different ratios of chitosan/Ox-QSG were applied with 75:25, 50:50, and 25:75, respectively. This work showed that oxidized-modified quince seed gum with chitosan has improved the thermodynamic, swelling properties, and degradation rate. SEM results proved that the elevation of contents of OX-QSG in the hydrogel provides a soft structure with lighter pore constructions attributed to the improved cross-link density resulting from the functional aldehyde of QSG. As an outcome, the elevated content of OX-QSG raises the number of pores (Figures 5a–5c). The porous structure is one of the most needed characteristics for hydrogel development, playing an important role in swelling (Ghorbani et al., 2020). Cell viability was also measured on NIH3T3 fibroblasts and demonstrated with CS/OX-QSG (25:75) hydrogel, providing suitable cell growth and survival with enhanced cell attachment (Figure 5d).

### 4.3. Scaffolds from other seed derivatives

**Figure 6:**
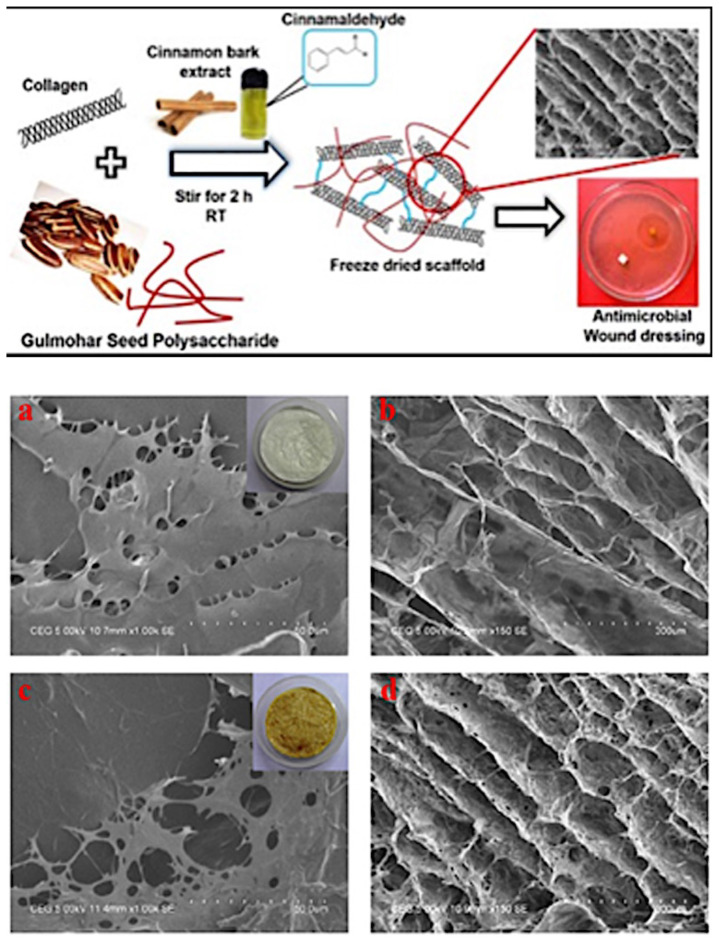
SEM images of (a) native collagen scaffold and its (b) cross-section morphology (100/0 wt.% C/GSP) and the hybrid scaffold (100/100 wt.% C/DSP), (c,d). Reproduced, with permission, from Cheirmadurai et al. (2016).

Besides gum and mucilage, seeds can also contain oils, fibers, and various other substances that can be used for a variety of purposes. These seed derivatives may also be used in scaffold fabrication to improve cell attachment, mediate mechanical behaviors, and eventually be used as a candidate scaffold for tissue regeneration. Galactomannan is a seed byproduct and acts as a storage polysaccharide. The family fabaceae is rich in galactomannan. Cheirmadurai et al. (2016) prepared hybrid collagen scaffolds comprising glumahor (*Delonix regia*) plant from seed polysaccharide (GSP) to study the wound healing efficacy of the extract (Cheirmadurai et al., 2016). The polysaccharide amount was mixed with different concentrations and increased by 25% of collagen (Figure 6). The thermodynamic characterization and the stability were improved in the hybrid scaffolds than in the one based on collagen only and this was attributed to the feeble connections from GSP. Native collagen has a relatively high pore distribution compared to the hybrid one, whereas the 100/100 wt.% Collagen/ GSP hybrid scaffold exhibits a film-like surface with a low pore distribution (Figures 6a and 6b). The porosity, permeability, and swelling properties were enhanced thanks to the structure of the hybrid scaffold (Figures 6c and 6d). This shows that the polysaccharide extracted may have the potential for deposition over the collagen. Besides chemical exchanges, the swelling ability, which affects cell behaviors such as cell adhesion, growth, and differentiation, was enhanced (Cheirmadurai et al., 2016). Enzymatic degradation and antimicrobial properties were also improved in the presence of GSP extract. It has been proposed that hybrid scaffolds, with their improved antimicrobial activity, can be used successfully in wound healing and skin scaffold applications.

Seeds may also contain various other substances that have medicinal or other uses, such as flavonoids, phytosterols, and antioxidants. In a study reported by Garcia et al. (2021), a nanohydroxyapatite (nHA) and collagen blended scaffold, along with plant extracts, were produced for bone tissue regeneration. Grape seed, pomegranate peel, and jabuticaba peel extracts were utilized as collagen crosslinker agents to improve the material’s properties. With these flavonoids, the mechanical properties of the scaffold were improved and showed better biocompatibility and antimicrobial activity (Garcia et al., 2021).

## 5. Conclusion

Plant seed derivatives such as mucilage and gums are sustainable and low-cost edible biopolymers. The valued functionality of these extracts is related basically to their chemical structure as carbohydrates. Several health benefits have been ascribed to those extracts besides their pharmaceutical, food-processing, and industrial applications such as film, emulsifiers, and gelling agents. The use of seeds in several industries can have a range of environmental, economic, and social impacts. For instance, the use of seeds in the pharmaceutical industry can lead to the development of novel, expensive medications, and treatments, which may have an impact on healthcare costs and treatment access; improving public health quality and contribute to economic growth in the areas where they are produced. However, a sustainable agricultural practice is a need for the standardization of the extraction and the production.

This review highlighted a summary of plant-seed-derived extract applications, mainly mucilage and gum, to develop functional scaffolds in different forms with various blend formulations, to be used in several tissue engineering applications. Features such as nontoxicity, antiinflammatory, biocompatibility, and tunable biomechanical property make those smart biopolymers more precious for developing functional scaffolds.

It is believed though that the future applications will be focusing on the total uses of these extracts in the biosynthesis and the processing of new delivery systems. Future prospective considers seed-derived extract as the most luminous part of tissue engineering and drug delivery, given that this prosperous composition is the crucial factor urging researchers to gear towards recovering their beneficial compounds for various applications apart from industrial and pharmaceutics uses, such as for rare diseases and cancer treatment considering the antioxidant, antiinflammatory, and anticancerous potential. In particular, the improvement in 3D printing technologies and applications and the fact that these seed-derived materials possess suitable properties for printing may increase the number of applications in this field.

## Figures and Tables

**Table 1:** Mucilage extraction techniques from various seed sources.

Extraction methods	Seed source	References

Chemical and enzymatic		

Acid	*Arabidopsis thaliana*	(Macquet et al., 2007)
Alkali	*Arabidopsis thaliana*	(Macquet et al., 2007)
Fermentation	*Theobroma cacao*	(Ramos et al., 2014)
	*Theobroma grandiflorum*	(Ramos et al., 2014)
Enzymes	Flaxseed5	(Wanasundara and Shahidi, 1997)

Physical		

Extracted by centrifugation	*Linum usitatissimum* L.	(Nayak et al., 2013)
Ultrasound	*Linum usitatissimum* L.	
Cold extraction	*Salvia hispanica*	(Tavares et al., 2018)
Thermal extraction	*Aegle marmelos*	(Jindal et al., 2013)
Microwave	*Cydonia oblonga*	(Yilmaz et al., 2021)
Supercritical fluids	*Lallemantia royleana*	(Falahati and Ghoreishi, 2019)

**Table 2:** Prominent seed-extract-based tissue scaffolds, their botanical sources, and pharmaceutical applications.

Fabricated construct	Source	Form	Role	Test model	Ref.
**Cryogels of silicon-integrated quince seed mucilage (Si-QSM)**	Quince seed (*Cydonia oblonga*)	Cryogel	Upregulation of osteogenesis-related genes	hAMSCs	(Yilmaz et al., 2021)
**Glucuronoxylan-based quince seed hydrogel**	Quince seed (*Cydonia oblonga*)	Hydrogel	Biocompatibility and NIH-3T3 cell viability and proliferation	NIH-3T3 cells	(Guzelgulgen et al., 2021)
**Collagen/Glumahor seed polysaccharide (C/GSP) scaffolds**	*Delonix regia* seed	Hybrid collagen scaffolds	Antimicrobial activity on wounds	*Bacillus subtilis Staphylococcus aureus and Escherichia coli*	(Cheirmadurai et al., 2016)
**Polyacrylamide grafted polymeric blend of fenugreek seed mucilage- Polyvinyl alcohol (FSM-PVA-g-PAM**)	Fenugreek seed (*Trigonella foenum-graecum*)	Powder/tablet	Drug delivery Controlled release of loaded enalapril maleate Cell growth and differentiation Wound healing	In vivo	(Bal and Swain, 2020)
**PCL/QSM/PEG polymer**	Quince seed (*Cydonia oblonga*)	Electrospun nanofibers	Human Adipose Mesenchymal Stem Cells (hAMSCs) adhesion wound dressings Supports fibroblasts growth	hAMSCs	(Urena-Saborio et al., 2018)
**Basil seed mucilage and polycaprolactone (BSM/PCL)**	Basil seed (*Ocimum basilicum*)	Electrospun nanofibers	Adhesion and growth of cells	Epithelial Vero cells	(Allafchian et al., 2018)
**Chitosan/oxidized-modified quince seed gum/curcumin-loaded in halloysite nanotubes (CS/OX-QSG/ CUR-HNTs)**	Quince seed (*Cydonia oblonga*)	Hydrogel	NIH3T3 fibroblast attachment	NIH3T3 cells	(Yavari Maroufi and Ghorbani, 2021)
**Basil seed mucilage–chitosan- Ziziphora clinopodioides essential oil-MgO nanoparticles**	Basil seed (*Ocimum basilicum*)	Films	Antimicrobial activity Increasing of shelf-life of stored food	*Staphylococcus aureus*, *Listeria monocytogenes*, *Bacillus cereus*, *and Bacillus subtilis*	(Naeeji et al., 2020)
**Nano-carrier ** ***Azadirachta indica*** ** seed-gum**	*Azadirachta indica* seed	Nanocarrier	Antioxidant and anticancer activity Synthesis of nanocarrier to carry curcumin anticancer drug Effective against MCF7 cancer cell line	MCF-7 cells	(Samrot et al., 2020)
**Asafoetida seed-gum silver nanoparticles**	Asafoetida seed (*Ferula foetida)*	Nanoparticle	Inhibiting the multiplication of MCF-7 cancer cells Cell toxicity and antimicrobial Antibacterial and antifungal activity	MCF-7 cells and *E. coli*, *K. pneumoniae*, *C. albicans*	(Devanesan et al., 2020)
**Polycaprolactone/quince seed mucilage**	Quince seed (*Cydonia oblonga*)	Electrospun nanofibers	Biocompatible scaffold environment Easy culture of epithelial Vero cells Providing scaffold with lower fiber diameters and higher hydrophilicity	Epithelial Vero cells	(Allafchian et al., 2020)
**Fe** **_3_** **O** **_4_** ** chitosan– bovine albumin serum-quince-seed mucilage (CS–BSA–QSM–Fe** **_3_** **O** **_4_** ** NPs)**	Quince seed (*Cydonia oblonga*)	Polymeric composite	Controlled protein release	*N/A*	(Hajivand et al., 2019)
**Plantago major seed mucilage/PVA nanofibers (PMM-PVA)**	Plantago major seed	Electrospun nanofibers	Improvement in mechanical strength and cell viability Epithelial cell growth	Vero cells	(Golkar et al., 2019)
**Polyurethane (PU)-grape seed oil-honey-propolis**	Grape seed	Electrospun nanofibrous composite	Cells better blood compatibility and cell viability rates of human fibroblast cells (HDF)	HDF cells	(Chao et al., 2018)
**Chitosan (CS)/silk sericin (SS)/β-glycerophosphate (β-GP) loaded with longan seed extract**	Longan seed (Dimocarpus longan)	Hydrogel	Fibroblast cell line (NCTC clone 929) and osteoblast cell line (MC3T3-E1) survival Attachment of MC3T3-E1 cells	MC3T3-E1 cells	(Pankongadisak and Suwantong, 2018)
***Phoenix dactylifera*** ** seeds incorporated chitosan/ hydroxyapatite**	*Phoenix dactylifera* seed	Nanocomposite	Superior in vitro biomineralization and biocompatibility Enhanced mechanical properties and osteoinductivity	Human osteoblast like MG-63 cells	(Jolly et al., 2020)
**Quince seed mucilage supplemented with titanium dioxide (TiO2) and silicon oxide (SiO2) nanoparticles**	Quince seed (*Cydonia oblonga*)	Films	Structural improvement Antibacterial activity	*Staphylococcus aureus, Bacillus subtilis, Bacillus cereus, Listeria monocytogenes Salmonella Typhimurium Escherichia coli*	(Shahbazi and Moosavy, 2019)
**Balangu (** ***Lallemantia royleana*** **) seed mucilage (BSM) loaded with paracetamol**	*Lallemantia royleana*	Aerogels	Paracetamol drug loading	*N/A*	(Falahati and Ghoreishi, 2019)
